# Targeting Hypoxia-Driven Metabolic Reprogramming to Constrain Tumor Progression and Metastasis

**DOI:** 10.3390/ijms21155487

**Published:** 2020-07-31

**Authors:** Marisol Miranda-Galvis, Yong Teng

**Affiliations:** 1 Department of Oral Biology and Diagnostic Sciences, Dental College of Georgia, Augusta University, Augusta, GA 30912, USA; mgalvis@augusta.edu; 2 Georgia Cancer Center, Department of Biochemistry and Molecular Biology, Medical College of Georgia, Augusta University, Augusta, GA 30912, USA; 3 Department of Medical Laboratory, Imaging and Radiologic Sciences, College of Allied Health, Augusta University, Augusta, GA 30912, USA

**Keywords:** hypoxia, metabolic reprogramming, tumor microenvironment, progression and metastasis, signaling network

## Abstract

Hypoxia in locally advanced solid tumors develops due to uncontrollable cell proliferation, altered metabolism, and the severe structural and functional abnormality of the tumor vasculature, leading to an imbalance between oxygen supply and consumption in the fast-growing tumors and negative impact on the therapeutic outcome. Several hypoxia-responsive molecular determinants, such as hypoxia-inducible factors, guide the cellular adaptation to hypoxia by gene activation, which is critical for promoting malignant progression in the hostile tumor microenvironment. Over time, a large body of evidence exists to suggest that tumor hypoxia also influences the tumor metabolic reprogramming, resulting in neoangiogenesis, metastasis, and immune evasion. In this respect, our review aims to understand the biological processes, key events, and consequences regarding the hypoxia-driven metabolic adaptation of tumor cells. We also assess the potential therapeutic impact of hypoxia and highlight our review by discussing possible therapeutic strategies targeting hypoxia, which would advance the current understanding of hypoxia-associated tumor propagation and malignant progression and improve the management of tumor hypoxia.

## 1. Introduction

Oxygen is indispensable for cellular metabolism in multicellular organisms. A set of metabolic reactions involving oxidative phosphorylation (OXPHOS) utilizes the oxygen to generate adenosine triphosphate (ATP), which is the molecular currency in the intracellular energy transfer and essential to develop almost all cellular and biological processes. No doubt, cells are provisioned with specialized chemoreceptors to sense increased (hyperoxia) or decreased (hypoxia) oxygen levels [1]. As a result, adaptive processes are stimulated during physiological conditions that compromise the oxygen environment (e.g., embryonic development and exercise), permitting tight control of the homeostasis [2,3]. Otherwise, decompensation in oxygen regulation may lead to the development and/or progression of several pathologic disorders, including inflammation, cardiovascular diseases, and cancer [4].

Tumor cells are mainly characterized by their ability to sustain chronic proliferation. The fast-paced growth demands modifications in the tumor metabolism to support the high rate of energy requirements [5]. Furthermore, the newly formed and dysfunctional vascular supply produces areas of tumor hypoxia that demand alternative metabolic pathways nondependent of oxygen [4]. The reprogramming energy metabolism by tumor cells has been recognized and well-characterized as one of the emerging hallmarks of cancer [5]. Nevertheless, the effect of hypoxia in the adjustment of cancer metabolism has recently gained significant attention, not only for the hypoxic areas that cause the prompt growing tumor but also for the hypoxic effect in the tumor microenvironment that produces the antiangiogenic factors [6]. In order to develop new therapeutic strategies that improve the efficacy of antineoplastic agents and overcome the treatment resistance, we take a detailed look at the biological processes, key events, and consequences regarding the hypoxia-driven metabolic adaptation of tumor cells.

## 2. Biologic Aspects of Tumor Hypoxia

Tumor hypoxia results from decreased microenvironment oxygen availability. Under physiological conditions, the average oxygen tension in normal tissues is approximately 40 mmHg [7]. The parameters to recognize the reduced oxygen levels may vary widely among several types of cancer. A general approach for solid tumors considers the tumor hypoxic thresholds in the range of 0.02 mmHg to 35 mmHg [8]. Tumor cells adapt to hypoxic conditions, inducing genomics, and proteomics modifications that lead to structural and functional alterations. A decreased proliferation rate is the main physiologic feature that distinct hypoxic from normoxic cells. Cells under hypoxic conditions switch to a quiescent, apoptotic, or even necrotic status [4]. Moreover, recent in vitro models recreating a microenvironment with oxygen restriction provide evidence that hypoxia can promote the expression of stem-cell-associated genes, inducing cancer stem cell properties [9]. Remarkably, the interruption of the proliferative signaling is compensated by the continuous induction of angiogenesis regulated mainly by the expression of vascular endothelial growth factor-A (VEGF-A) [10]. Thus, hypoxic niches generate a heterogeneity population of malignant cells that contribute to the aggressiveness of progression and spread.

On the flip side, the intratumoral microenvironment may also exhibit different oxygen concentrations along tumor regions for a variety of lapse time, resulting in the classification of tumor hypoxia into the three categories acute, cycle, and chronic.

### 2.1. Acute Hypoxia

Acute hypoxia (also known as perfusion-limited hypoxia) is the local abrupt, temporary, and reversible disruption in the flowing blood, producing oxygen fluctuation in the tumor microenvironment [11]. It occurs as a result of the severe structural and functional abnormality of the tumor vasculature. The blood vessels are immature, tortuous, hypermutable, over dilated, and disrupted [12]. The dual pathophysiology of acute hypoxia allows subcategorization into two subgroups. Hypoxemic hypoxia is produced by a severe reduction in the pressure of oxygen, while ischemic hypoxia is the consequence of temporal embolism caused by blood clots or vascular occlusion by cell aggregates, and high interstitial pressure [13]. Analysis of experimental models mimicking the hypoxic environment has allowed a significantly advanced understanding of cellular and molecular biology of hypoxia. Whereas studies using tumor cell lines show that acute hypoxia is induced in the time frame of 30 min to 72 h, in vivo experiments recreates perfusion-limited hypoxic conditions using periods of an oxygen-deprived environment of 2 to 4 h every day or continuous for 28 days [14,15].

### 2.2. Cyclic Hypoxia

Intermittent blood flow consequence of the sprouting of disorganizing and defective blood vessels, generate cyclic changes of oxygen concentrations, resulting in a microenvironment with alternating states of hypoxia and reoxygenation [16]. Cyclic hypoxia, also called intermittent hypoxia, has been recreated in vitro assays in the variate time frame to 2–12 cycles of 10 min to 1 week of hypoxia and 10 min to 1–3 weeks of normoxia. Besides, studies in animal models used 1–12 cycles of 1 h of hypoxia and 1 h of normoxia [17].

### 2.3. Chronic Hypoxia

**Figure 1:**
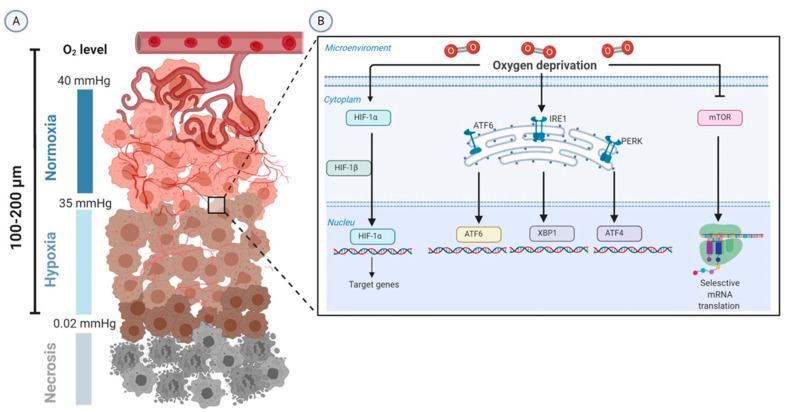
Schematic representation of the biology of tumor hypoxia. (**A**) The severe structural and functional abnormality of the tumor vasculature and the increase in diffusion distances between tumor cells and blood vessels (100–200 µm) lead to intratumoral hypoxia (oxygen tension 0.02–35 mmHg). (**B**) HIF-1α is the molecular determinant that is upregulated in the adaptive responses to alterations in tissue oxygenation. Moreover, tumor hypoxia induces adaptive responses through enhancing unfolded protein response (UPR)-mediated upregulation of ATF6, XBP1, and ATF4 and inactivating the mTOR pathway. Abbreviations: ATF, activating transcription factor; HIFs, hypoxia-inducible factors; IRE1, inositol-requiring protein 1; mmHg, millimeter of mercury; mTOR, kinase mammalian target of rapamycin; O2, oxygen; PERK, protein kinase RNA-like endoplasmic reticulum (ER) kinase; XBP1, X-box binding protein.

Chronic hypoxia arises from the long-term insufficient oxygenation and several conditions are capable of producing it. An increased diffusion distance between tumor cells and blood vessels from 100 to 200 μm, or about 10–20 cell layers, is the main cause of diffusion-limited hypoxia [18,19]. The other two recognized subtypes of chronic hypoxia that occur to a lesser extent are the hypoxemic hypoxia and hypoxia due to compromised perfusion of leaky microvessels [13]. Experimental studies culturing tumor cell lines replicate chronic hypoxic conditions using a time frame from 4 h to 8 weeks [17] (Figure 1A).

## 3. Cellular Adaptation to Hypoxia

Hypoxia-inducible factors (HIFs), a highly conserved class of transcription factors, are the master molecular regulators in the adaptive responses to alterations in tissue oxygenation [20]. Furthermore, emerging evidence has highlighted the important role played by the kinase mammalian target of rapamycin (mTOR), the unfolded protein response (UPR) in the tumor biology controlled by oxygen deprivation (Figure 1B) and nuclear factor-κB (NF-κB).

### 3.1. Hypoxia-Inducible Factor 1-Alpha (HIF-1α)

HIF-1 is a transcriptional heterodimer complex, composed of two basic helix-loop-helix-PAS (bHLH-PAS) domains, the signal-regulated subunit α (HIF-1α) and the unregulated subunit β (aryl hydrocarbon receptor nuclear translocator-ARNT, also known as HIF-1β). The human *HIF-1α* gene is located on chromosome 14 (q23.2) and consists of 16 exons coding for HIF-1α protein, whereas the human *ARNT* gene is located on chromosome 1 (q21), with 22 exons coding for ARNT protein. While HIF-1α stability, intracellular localization, and activity, are regulated by the cellular oxygen levels, ARNT is constitutively expressed in the nucleus [21]. Nevertheless, emerging evidence demonstrated that ARNT protein may also be upregulated through an oxygen deprivation pathway in specific tumor cells, such as melanoma, cervix adenocarcinoma, hepatoma, breast carcinoma, and prostate cancer [22].

The bHLH and the two PAS domains, PAS-A and PAS-B, represent the main structural features of both subunits and are essential for DNA binding and heterodimerization. ARNT protein is crucial for ligand-binding with the hypoxia response elements (HREs) in the DNA, allowing the nuclear translocation from the cytosol through the importin α/β-dependent pathway [22]. In addition, this protein carries a transactivation domains (TAD) that are composed of the NH2-terminus (N-TAD, exclusively in α subunit) and COOH-terminus (C-TAD). Distinct from ARNT protein, HIF-1α comprises an oxygen-dependent degradation domain (ODDD), that overlaps with N-TAD and regulates the protein stability. Under normoxic conditions, this region is responsible for the hydroxylation of HIF-1 through the pVHL-mediated ubiquitin-proteasome pathway. The enzymes involved in this catalytic reaction are prolyl-hydroxylases (PHDs), which use oxygen, α-ketoglutarate, and ferrous iron to hydroxylate HIF1α. Thus, low oxygen levels stabilize and accumulate HIF-1α protein, allowing the translocation to the nucleus and the formation of the transcriptional active heterodimer [20,23]. Early evidence suggested that PHD2 was the main enzyme able to suppress the activity of fusions containing the CODD prolyl hydroxylation site. However, other PHD family members, such as PHD3, also contribute to the downregulation of the HIF pathway in a nonredundant manner [24]. On the flip side, C-TAD interacts with p300/CBP to be responsible for modulating the gene transcription and protein activity through the oxygen-dependent factor inhibiting HIF (FIH). It is important to recognize that HIF-1α function and stabilization are regulated by some other cellular pathways involving oxygen-independent mechanisms, such as growth factors, cytokines, and reactive oxygen species (ROS) [25].

Among the HIF proteins, HIF-1α has been more characterized and discovered the signaling pathways involved in the cancer progression, which is due to its high overexpression in most of the solid tumors and their metastases [26]. HIF-1α activation influences every critical aspect of tumor development, progression, and metastasis, through the transcription of more than 1000 genes that encode key proteins involved in genetic instability, immortalization, cell proliferation, immune evasion, angiogenesis, metabolic reprogramming, invasion, and treatment resistance [27]. The expression of several growth factors that regulate tumor growth after hypoxia injury, such as insulin-like growth factor (IGF2), transforming growth factor (TGF), and platelet-derived growth factor (PDGF), are under HIF-1α regulation [4]. Remarkably, HIF-1α impacts two of the more essential processes for tumor development and progression, angiogenesis and metabolism. While the new blood vessels formed through the expression of the transcription of *VEGF* proportionate a supportive microenvironment with oxygen and nutrients, the glucose metabolic switch allows adjustments in the acquisition of energy [28].

### 3.2. The mTOR Pathway

mTOR (also known as FRAP) is a serine/threonine protein kinase belonging to a family of six phosphatidylinositol 3-kinase-related kinases (PIKKs) proteins. mTOR assembles two different structurally and functionally complexes, mTORC1 and mTORC2. Although mTORC1 is well-characterized, only a few studies have recently described the biological function of mTORC2. mTORC1 is conformed by mTOR, a regulatory-associated protein of mTOR (Raptor), mammalian lethal with Sec13 protein 8 (mLST8), proline-rich AKT substrate 40 kDa (PRAS40), and DEP-domain-containing mTOR-interacting protein (Deptor) [29]. mTOR is a core regulator of several biological processes, such as the regulation of protein synthesis, cell growth, cell proliferation, cell motility, apoptosis, and autophagy. Given those crucial functions, it is perhaps not surprising that mTOR is one of the most frequently affected pathways in human cancers. Dysfunctions in mTORC1 and/or mTORC2, mutations in the *mTOR* gene, and/or aberrations in signaling cascade, results in hyperactivation of the pathway, alterations in downstream suppressor genes and oncogenes, that contribute to the cell malignant behavior [30].

Microenvironmental availability of oxygen, together with other essential nutrients (e.g., glucose, amino acids, lipids, growth factors, and cytokines) allows mTORC1 activation. mTORC1 activity triggers the phosphorylation of p70S6 kinase (p70S6K), eukaryotic initiation factor 4E binding protein 1 (4E-BP1), and eukaryotic elongation factor 2 kinase (EEF2K). Thus, mTOR is downstream of other factors that sense oxygen, and decreased oxygen levels have the potential to inhibit the function of mTOR in cells [31]. Intratumoral hypoxia has the ability to downregulate or inactive mTORC1 in three different ways, of which two involve the initiation of its negative regulator TSC1-TSC2 (hamartin–tuberin) complex. The activation of the TSC1-TSC2 complex may occur via the activation of AMP-activated protein kinase (AMPK) [32] or regulated in development and DNA damage responses 1 (REDD1) [33]. The third mechanism blocks the interaction mTOR-RHEB through the proteins Bcl2/adenovirus E1B 19-kDa protein-interacting protein 3 (BNIP3) [33], and promyelocytic leukemia protein (PML) [34]. The negative regulations of mTOR mediated by hypoxia are important in tumorigenesis due to its role in maintaining the synthesis of proteins that are essential for cell survival even in oxygen deprivation [35].

### 3.3. UPR

The biosynthesis, folding, modification, and transport of proteins occurs in the endoplasmic reticulum (ER) under a supervised process highly sensitive to microenvironment changes such as glycosylation, redox status, and availability of glucose, calcium, and oxygen. Consequently, the ER is provided with stress sensors including inositol-requiring protein 1 (IRE1), protein kinase RNA-like ER kinase (PERK), and activating transcription factor 6 (ATF6), that stimulate by binding immunoglobulin protein (BiP) activate a defense mechanism termed UPR [36]. This complex signal transduction pathway transmits information about the alterations in protein folding to the cytoplasm and the nucleus, producing transcriptional activation of UPR target genes, the rapid translational arrest of global protein synthesis, and endoplasmic reticulum-associated degradation. Despite these mechanisms intended to reestablish the cell viability and function, if the homeostasis cannot be achieved cells switch the process of programmed cell death under physiological conditions [35].

Research over the past decades has demonstrated that the deregulation of the components of UPR participates in the multi-step process of carcinogenesis, tumor progression, and metastasis. While PERK has been linked with tumor growth and progression, ATF6 is related to metastasis, and IRE1 contributes to almost all the hallmarks of cancer [37]. Interestingly, tumor hypoxia induces adaptive responses via controlling protein synthesis mediated by UPR. Phosphorylation of eukaryotic initiation factor 2 alpha (EIF2α), through activation of PERK, downregulates global protein translation while selectively allowing the expression of activating transcription factor 4 (ATF4) [38]. Products of these events promote hypoxia tolerance, allowing cell survival and protection from apoptosis and tumor growth [39]. Moreover, tumor cells exposed to hypoxia upregulate X-box binding protein (XBP1), a transcription factor for several oncogenic genes [40].

### 3.4. NF-κB

Besides the mentioned above, the response to the cell stress generated by the hypoxic conditions involves, to some extent, the main inflammatory inductive transcription factor, NF-κB [41]. The activation of NF-κB is regulated by the canonical pathway via the IκB kinase complex (IKK) [42]. Recently, chronic hypoxia has been linked to the upregulation of the NF-κB signaling cascade through the enzymes PHDs and FIH [41].

Strikingly, the crosstalk between HIF (hypoxia) and NF-κB (inflammation) has been discovered, which includes common activating stimuli, and shared regulators and targets [43]. At a first glance, NF-κB is a direct basal modulator of the *HIF* gene expression [44], then HIF in turn stimulates the expression of alarmin receptors to trigger the NF-κB signaling [45]. Over the last years, it was recognized some physical interactions among HIF subunits and NF-κB, including HIF-1α-RelA, HIF-1β-RelB/p52, and HIF-2α-IKKγ. Besides common regulators (e.g., TRAF6, FBW7, and VHL), HIF and NF-κB target a large number of common genes (e.g., *VEGF*, *BNIP3*, and *IL-*8) as transcription factors [43].

The crosstalk between HIF- and NF-κB-mediated signaling, apart from their physical and functional interaction, plays a critical role in regulating a large array of genes involved in every critical step of carcinogenesis, tumor progression, metastasis and resistance of cancer therapy [46].

## 4. Genetic and Metabolic Modifications in the Hypoxic Tumor Microenvironment

Tumor hypoxia through HIF-1α regulation drives genetic instability in a large group of genes that determine malignant and aggressive molecular features. Failures in DNA damage repair alter cell cycle checkpoints and chromosomal aberrations, leading to genomic changes related to deficiencies in oxygenation [39]. In turn, numerous genes that encode metabolites and enzymes involved in metabolic processes contribute to tumor metabolic reprogramming.

Oxygen is an indispensable factor for aerobic metabolism. After glucose breakdown, the pyruvate into the tricarboxylic acid cycle (TCA), and then the oxygen is used as a terminal electron acceptor in the OXPHOS to produce ATP. Besides ATP, the set of metabolic reactions generates carbon dioxide and a molecule of water. Adaptive responses to deficiencies in oxygenation allow the cells to significantly reduce the amount of pyruvate, which transfers to the mitochondria and switches from OXPHOS to substrate phosphorylation to guarantee the ATP production. The metabolic reaction generates lactic acid as a waste product instead of carbon dioxide [5]. The alternative metabolic pathways confer tumor cells to survive and grow in the microenvironment with oxygen deprivation. Interestingly, tumor cells prefer substrate phosphorylation over OXPHOS even in the conditions with normal levels of oxygen due to this pathway providing cancer cells with important advantages. This process is so-called Warburg effect, known as aerobic glycolysis [40]. Despite aerobic metabolism being more productive generating 32 molecules ATP/mol glucose versus two molecules ATP/mol glucose createds through OXPHOS, increased glycolysis flux produces higher amounts of ATP in a shorter time frame [47]. It supplies the high demands of energy during tumorigenesis, tumor progression, and metastasis.

The metabolic intermediates produced in the glycolysis are used in the macromolecular biosynthesis of proteins, lipids, and amino acids, contributing not only to sustain the high rate of cell proliferation but also to the clearance of metabolic waste products. Lactate recycling occurs in surrounding normoxic cells which express monocarboxylate transporter 1 (MCT1) to import lactate and convert it to pyruvate for the mitochondrial metabolism [48]. Fatty acids are other essential substrates of tumor cells as they are the major component of cell membranes, facilitating the membrane fluidity, and maintaining mitochondrial activity. The mitochondrial aerobic process of breaking down a fatty acid into acetyl-CoA units is so-called fatty acid oxidation, which produces energy to fuel the cell [49]. Hypoxia, via the transcriptional machinery of HIF-1α, modulates the reprogramming of lipid metabolism, resulting in the support of fast tumor proliferation. In order to enhance lipogenesis, HIF-1α induces the transcription of genes involved in fatty acid uptake (e.g., *PPARγ*, *FABP3*, *FABP4*, and *FABP7*), endocytosis of lipoproteins (e.g., *LRP1* and *VLDLR*), and fatty acids synthesis (e.g., *SREBP-1* and *FASN*). Moreover, HIF-1α activates target genes involved in lipid accumulation (e.g., *PLIN2* and *HIG2*) and inhibition of lipolysis (e.g., *ATGL*), once fatty acids are converted into neutral triacylglycerols (e.g., *AGPAT2* and *LIPIN-1*) [50].

HIF-1α plays a major role in the glycolytic switch in two different ways, including the regulation of genes encoding glycolytic enzymes involved in the promotion of aerobic glycolysis and the decrease in the mitochondrial functions.

### 4.1. Promotion of Anaerobic Glycolysis

Facilitative glucose transporters (GLUTs) are a family of 14 membrane proteins responsible for extracellular glucose import. Tumor cells require a high amount of glucose, not only for the prompt rate of proliferation but also for the low efficiency of aerobic glycolysis. HIF-1α binds to *cis*-acting binding sites of the *GLUT1* and *GLUT3* genes inducing the overexpression, and as a result, the rate of import glucose is increased [51,52].

HIF-1α promotes the expression of crucial glycolytic enzymes hexokinase 2 (HK2) and phosphofructokinase 1 (PFK1), producing the acceleration of the glycolytic flux at different levels. HK2 phosphorylates the intracellular glucose to glucose 6-phosphate (G6P) into the investment phase [53]. PFK1 catalyzes the important control point of converting fructose 6-phosphate (F6P) to fructose 1,6-bisphosphate (FDP) and ADP, and it is regulated allosterically [54,55]. The promoter activities of *PFK1* are upregulated through direct binding of HIF-1α in the gene promoter region [56]. Interestingly, PFK1 is encoded by the gene *6-phosphofructo-2-kinase/fructose-2,6-biphosphatase 2* (*PFKFB2*) that is also induced by HIF-1α. [57]. Another direct target gene of HIF-1α is *lactic dehydrogenase A* (*LDHA*). HIF-1α interacts with the HRE-D site in the *LDHA* gene promoter to induce its expression [58]. LDHA then participates in the last step of glycolysis, catalyzing pyruvate into lactate with concomitant generation of nicotinamide adenine dinucleotide (NAD^+^) from NADH [59].

**Figure 2:**
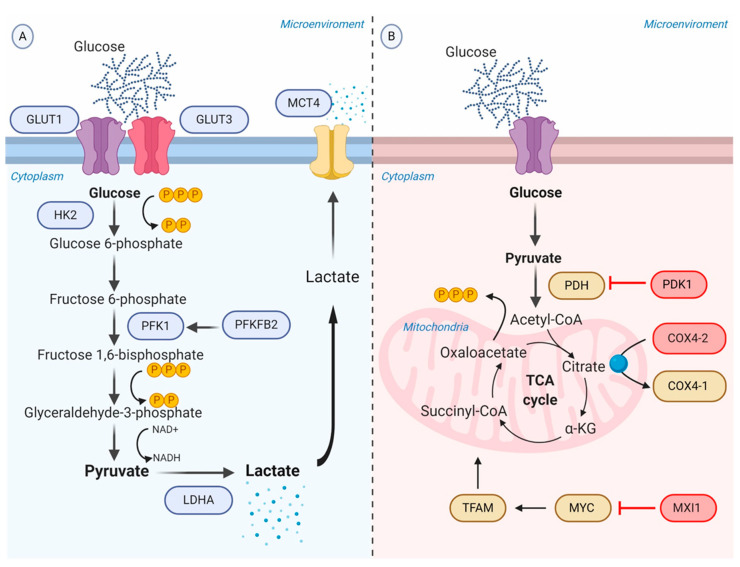
Schematic representation of the glycolytic switch mediated by tumor hypoxia. (**A**) Regulation of genes encoding glycolytic enzymes involved in the promotion of anaerobic glycolysis. GLUT1 and GLUT3 overexpression increase the rate of glucose import. HK2 phosphorylates the intracellular glucose to glucose 6-phosphate. PFK1, produced by PFKFB2 or induced by HIF-1α, converts Fructose 6-phosphate into Fructose 1,6-bisphosphate and ADP. LDHA catalyzes pyruvate into lactate with concomitant generation of NAD+ from NADH. MCT4 transports lactate to the extracellular environment. (**B**) Repression of OXPHOS. PDK1 inactivates PDH and blocks the conversion of pyruvate to acetyl-CoA. MXI1 inhibits the transcriptional activity of MYC and thereby inactivates MYC target genes (e.g., TFAM) involved in OXPHOS and mitochondrial dynamics, replication, and biogenesis. The generation and accumulation of ROS are controlled by the activation of the mitochondrial proteases COX4-2, responsible for the degradation of the COX4-1 isoform. Abbreviations: ADP, adenosine diphosphate; COX, cytochrome c oxidase; GLUTs, facilitative glucose transporters; HK2, hexokinase 2; LDHA, lactic dehydrogenase A; MCT, monocarboxylate transporter; MXI, MAX interactor; NAD+, nicotinamide adenine dinucleotide; OXPHOS, oxidative phosphorylation; PDH, pyruvate dehydrogenase; PDK1, Pyruvate dehydrogenase kinase; PFK1, phosphofructokinase-1; PFKFB2, 6-phosphofructo-2-kinase/fructose-2,6-biphosphatase 2; ROS, reactive oxygen species; TFAM, transcription factor A, mitochondrial.

As a consequence of the increased rate of aerobic glycolysis, the lactate synthesis rises and HIF-1α allows to regulate the intracellular acidification through the expression of the transmembrane protein MCT4, aimed to transport lactate to the extracellular environment [60]. The extracellular lactate coupled with the overexpression of carbonic anhydrase IX and XII (CA-IX and CA-XII) regulated by HIF-1α, promote the acidification of the tumor microenvironment ranging from 6.0 to 6.5. It is important to recognize that the acidic microenvironment under oxygen availability may also upregulate CA-IX independent of the HIF-1α pathway, though sharing the same transcriptional factors induced by HIF-1α [61] (Figure 2A).

### 4.2. Repression of Oxidative Phosphorylation

Pyruvate dehydrogenase (PDH) is the mitochondrial enzyme responsible for the conversion of pyruvate to acetyl-CoA to consequent entry into the TCA cycle. HIF-1α induces the expression of *pyruvate dehydrogenase kinase 1* (*PDK1*) gene encoding a kinase enzyme, which phosphorylates and inactivates PDH. Therefore, it represses OXPHOS and prevents aerobic respiration [62].

On the other hand, HIF-1α has the potential to inhibit mitochondrial metabolism through binding and transactivating the *MAX interactor-1* (*MXI1*) gene. The protein encoded by this gene competes for MAX binding and inhibits the transcriptional activity of c-MYC function, and thereby inactivating c-MYC target genes involved in OXPHOS and mitochondrial dynamics, replication, and biogenesis [63]. For example, MXI1 can downregulate transcription factor A, mitochondrial (TFAM), which is essential for transcription, replication, and packaging of mtDNA into nucleoids [64]. Moreover, HIF-1α controls the mitochondrial function in hypoxic conditions via activating the mitochondrial proteases cytochrome oxidase 4-2 (COX4-2), which degrades the COX4-1 isoform to protects the cell from the harmful accumulation of ROS [65] (Figure 2B).

## 5. Impact of Metabolic Reprogramming Driven-Hypoxia on Tumor Progression

Research in vitro and in vivo assessing tumor metabolic reprogramming driven by hypoxia continues to bring surprises to reveal not only its influence in the cell survival and tumorigenesis, but also in the tumor progression through immune escape, angiogenesis, metastasis, and radiotherapy and chemotherapy resistance.

Hypoxia-driven immunometabolic alternations have recently gained significant attention due to its capacity to downregulate the antitumor immune activity in various ways [66]. HIF-1α upregulates the transcription of genes encoding key glycolytic enzymes such as aldolase A (ALDA), phosphoglycerate kinase 1 (PGK1), and pyruvate kinase M (PKM), stimulating the glycolytic metabolism in immune cell populations [67]. In the first instance, it can promote the differentiation of highly glycolytic cells. However, the increased flux of glucose intake by tumor cells decreases the availability of extracellular glucose, which is required for immune cells for the ATP production and the synthesis of lipids, amino acids, and nucleotides [68]. Thus, glucose deficiencies may lead to immune cell dysfunction. High concentrations of glycolytic metabolites and the expression of cytokines and chemokines (e.g., CCL5, CXCL12, and CXCR4) driven by HIF-1α also generate an unfavorable scenario for immune cell recruitment. Indeed, an oxygen-deprived tumor microenvironment influences the migration and activity of both innate and adaptive immune cells [69].

The functions of dendritic cells (DCs) involve the presentation of tumor-derived antigens to naïve T cells to initiate the immune adaptive response. Hypoxia does not hamper DC maturation; however, in vitro assays support its important role in DC dysfunction through interfering with DC differentiation, adaptation, and activation. Hypoxia restricts the ability of immature DCs to capture antigens through the downexpression of Rho GTPase and ERM proteins. Moreover, DCs in response to low oxygen levels alter the expression of proinflammatory and proangiogenic cytokines [70].

The cascade of molecular events triggered by HIF-1α activation involves the secretion of cytokine and chemokines that attract monocytes, allowing for the differentiation into macrophages or tumor-associated macrophages (TAMs). TAMs represent the major inflammatory component of the tumor microenvironment and generate mechanisms to support the immune escape, such as the expression of the ligand-receptor PD-L1 that inhibits T cells cytotoxic function [71]. In addition, the concurrent inflammation induced by macrophages contributes to supporting the hallmark capabilities of tumor cells [5].

**Figure 3:**
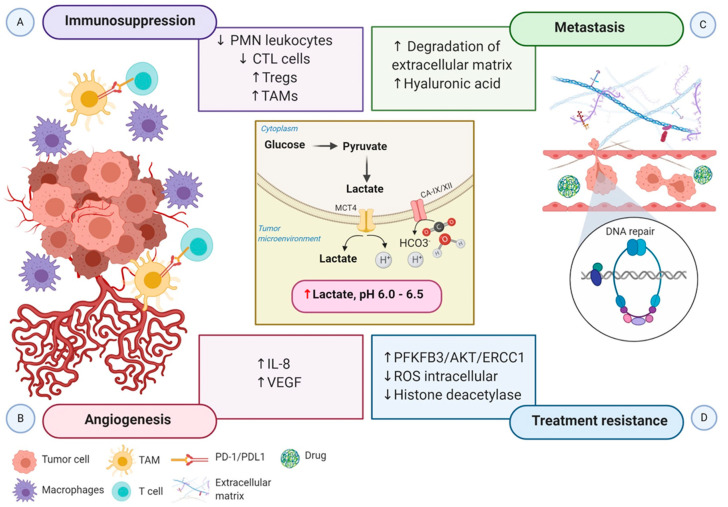
Schematic representation of the impact of hypoxia-associated metabolic reprogramming in tumor progression. The increased production of lactate along with the overexpression of CA-IX and CA-XII are regulated by HIF-1α directly, promoting the acidification of the tumor microenvironment. (**A**) The acidic microenvironment decreases the proliferation of polymorphonuclear leukocytes and CTL cells and increases the recruitment of Tregs cells. Moreover, the secretion of cytokines and chemokines attracts monocytes, enabling them to differentiate into macrophages or TAMs. TAMs express ligand-receptor PD-L1 that inhibits T cells cytotoxic function, and macrophages induce inflammation. Altogether, these mechanisms generate an immunosuppressive tumor microenvironment. (**B**) Elevated lactate in the tumor microenvironment boosts IL-8 and VEGF to facilitate angiogenesis. (**C**) Increased angiogenesis coupled with the acidic tumor microenvironment induces the degradation of the extracellular matrix and the expression of hyaluronic acid, which supports tumor cell migration and metastasis. (**D**) Lactate efflux activates metabolic substrates and the transcription of genes involved in metabolic rewiring to influence the tumor’s ability to respond to radiotherapy and chemotherapy-induced DNA damage. Increased PFKFB3 improves the ability of DNA repair, and reduced intracellular ROS accumulation increases the tolerance to DNA damage. Moreover, inactivation of histone deacetylase caused by elevated lactate promotes DNA repair. Abbreviations: CA, carbonic anhydrase; CTL, cytotoxic T lymphocytes; IL-8, interleukin 8; PD-1, programmed cell death protein 1; PD-L1, programmed death-ligand1; PFKFB3, 6-phosphofructo-2-kinase/fructose-2,6-biphosphatase 3; PMN, polymorphonuclear leukocytes; ROS, reactive oxygen species; TAMs, tumor-associated macrophages; Tregs, regulatory T cells; VEGF, vascular endothelial growth factor.

The acidic tumor microenvironment and the production of free oxygen radicals further reduce the migration and activity of polymorphonuclear leukocytes and cytotoxic T lymphocytes (CTLs). CTLs play a critical role in adaptive immune response promoting apoptosis in recognized tumor cells. Lack of CTL activity results in an immunosuppressive tumor microenvironment. Otherwise, hypoxia stimulates the recruitment of the immunosuppressive T cells regulatory (Tregs) through CCL28-CCR10, suppressing T-effector cells, and secreting VEGFA, which stimulate the tumor immune evasion [72] (Figure 3A).

The hypoxia and HIF-1α pathways have been shown to regulate angiogenesis through the transcription of genes involved in angiogenesis, as well as factors antiangiogenic. Human endocrine gland derived *VEGF*, *endoglin* (*ENG*), *leptin* (*LEP*), *low-density lipoprotein receptor-related protein 1* (*LRP1*), and *TGF-β3* are the HIF-1α target genes that are implicated in different steps in angiogenesis [73].

Growing evidence indicates that tumor metabolic reprogramming driven by hypoxia also participates in angiogenesis via HIF-1α independent pathway. Elevated lactate accumulation into the extracellular matrix (ECM) resulted from the accelerated aerobic glycolysis promotes the accumulation of two potent promoters of angiogenesis, interleukin 8 (IL-8) and VEGF/VEGFR2 signaling pathway [74]. MCT1 increases the lactate influx from the ECM to inside of endothelial cells, triggering IL-8 expressions through the NF-ĸB signaling pathway, in a ROS and IĸBα-dependent manner [75]. Moreover, lactate stimulates tyrosine phosphorylation of the receptors tyrosine kinases Axl, Tie2, VEGFR-2, and ephrin type-A receptor 2 (EphA2), leading to the stimulation of the PI3K/Akt pathway in endothelial cells. Altogether, high levels of lactate potentiate the proliferation of endothelial cells, modulate the endothelial phenotype, and the neovessel formation, supplying the tumor with a supportive microenvironment [76] (Figure 3B).

Notably, HIF-1 participates in every critical aspect of cancer biology. While increased glycolytic metabolism empowers cancer cells to survive and evade immune attack, the molecular regulation of the epithelial to mesenchymal transition (EMT) and the new blood vessels confer malignant cells to invade and disseminate from the primary tumor to distant sites. Metabolic switching of tumor cells under hypoxic conditions, characterized by microenvironment acidification, also contributes to supporting tumor cell migration and metastasis. Lactic acid increases cancer cell motility, induces the synthesis of hyaluronic acid (HA) and the degradation of the ECM, and acts as a “natural selection regulator” that selective the most malignant phenotype profile of tumor cells [77,78] (Figure 3C).

Much of the interest in tumor metabolic network reprogram derives from its clinical impact on treatment resistance. The molecular mechanisms in the accelerated aerobic glycolysis and lactate efflux involve the transcription of genes, metabolic substrates, and events that influence the tumor responses to radiotherapy and chemotherapy at different levels. First, *6-phosphofructo-2-kinase/fructose-2,6-biphosphatase 3* (*PFKFB3*), a key gene of aerobic glycolysis, improves the ability of DNA repair through the PFKFB3/AKT/ERCC1 signaling pathway [79]. Second, the repression of OXPHOS leads to the accumulation of intracellular ROS and increasing the tolerance to DNA damage. At last, lactate inactivates the class I and II histone deacetylases (HDAC), resulting in the hyperacetylation of histones H3 and H4, alteration of chromatin compactness, promotion of DNA repair gene upregulation, and initiation of the nuclear activation of DNA-dependent protein kinase, catalytic subunit (DNA-PKcs) [80]. In addition, experimental evidence in tumor xenografts demonstrated that elevated lactate levels are correlated with a decreased response to fractionated irradiation, perhaps due to the antioxidative capacity of lactate [81] (Figure 3D).

HIF-1α and HIF-2α are responsible for the transcriptional activity of some genes involved in the maintenance and evolution of cancer stem cells (CSCs), such as POU domain, class 5, transcription factor 1 (POU5F1) [82], delta-like 1 Homologue (DLK1) [83], CD133 [84], and CD24 [85]. Despite the low prevalence of CSCs (1–3%), this subpopulation has a high resistance to conventional therapy due to its ability to self-renewal, differentiation, and tumorigenicity. Recent evidence indicates that hypoxic niches modify the CSC and promote the EMT-like phenotype under the deregulation of phosphatase and tensin homolog (PTEN) [86]. Interestingly the metabolic switch induced for hypoxia also contributes to it regulating certain genes related to CSCs, through increased secretion of exosomes [87].

## 6. Therapeutic Strategies Targeting Hypoxia

As previously discussed, a broad spectrum of solid tumors is characterized by a hypoxic microenvironment due to the fast tumor growth and the formed irregular microvascular network. Hypoxic niches generate a heterogeneity population of malignant cells contributing to the aggressiveness of progression and spread, in addition to reducing the response to antineoplastic therapies. As the central mediator of hypoxia, HIFs represent attractive and promising targets to overcome treatment resistance. Thus, over the last years, many HIF inhibitors have been developed to target the HIF pathway at a particular level (e.g., HIF mRNA expression, translation, stabilization, dimerization, DNA binding, transcriptional activity) or multiple levels. Nevertheless, HIF inhibitors in clinical trials and assessment have not shown efficacy, and until now they have yet been approved [88,89]. Moreover, the nuclear translocation of the HIF-1 heterodimer complex components represents an emerging targetable pathway [90]. Ivermectin, a specific inhibitor of the nuclear import importin α/β, has demonstrated to be effective to decrease the binding activity of HIF-1α to the importin α/β-heterodimer, significantly reducing the HIF-1α protein levels and the HIF transcriptional activity [91]. Along with other nuclear import inhibitors, nuclear export inhibitors are also explored and proposed as new opportunities for targeted therapy to treat hypoxic tumors, and some of which are currently in clinical trials [92]. Therapeutic strategies for the treatment of hypoxia tumors also include an inhibition/modulation of NF-κB activity. Several classes of NF-κB inhibitors that directly (such as aspirin) or indirectly (by blocking alarmin receptors) disrupt the NF-κB pathway, are currently being tested in conjunction with chemotherapy and radiotherapy.

Due to the rapidly evolving field of cancer biology, the understanding of the molecular mechanisms underlying hypoxia-driven reprogramming metabolism, and its impact on tumor progression, have led to propose new strategies to improve the efficacy of therapies targeting hypoxic tumor cells. Thus, the discovery of molecules interfering with the main process of the metabolic switch has shown interesting results in terms of controlling the fast-growing tumors. The first approach involves the regulators of pH, such as CAI-IX, MCT1, and MCT4. CA-IX-specific small molecule inhibitors (e.g., SLC-0111, DTP-348), and MCT1 and MCT4 small molecule inhibitors (e.g., AZD3965), have been used to target the catalytic activity of CA-IX and MCTs, while monoclonal antibodies against CA-IX (e.g., cG250) are used alone or in combination with other therapies. The second approach targets the enzyme LDHA in different ways: (1) inhibiting the gene expression (e.g., siRNA and shRNA against the *LDHA* gene); (2) affecting the enzyme activity (e.g., Compound 24, QLYNL, Oxamate); (3) modifying the pyruvate binding site (e.g., PSTMB); and (4) binding to the free enzyme (e.g., Galloflavin) [61,93].

## 7. Conclusions

A newly recognized hallmark of cancer is the reprogramming of energy metabolism directed to support the continuous and rapid cell growth and proliferation. Hypoxia exerts crucial functions in tumor metabolic reprogramming through hijacking multiple signaling pathways that fuel the increasing demand for the synthesis of biological molecules required for tumor development, progression, and treatment resistance. In this respect, targeting tumor metabolic rewiring, especially under the hypoxic microenvironment, is a key to successful tumor control. In the future, deciphering the mechanisms of hypoxia-associated tumor metabolic reprogramming in depth with the ultimate goal of informing the deployment of pathway inhibitors in hypoxic cancers is warranted.

## Abbreviations

4E-BP1	Eukaryotic initiation factor 4E binding protein 1
ALDA	Aldolase A
AMPK	AMP-activated protein kinase
ARNT	Aryl hydrocarbon receptor nuclear translocator
ATF4	Activating transcription factor 4
ATF6	Activating transcription factor 6
BiP	Immunoglobulin protein
BNIP3	Bcl2/adenovirus E1B 19-kDa protein-interacting protein 3
CA-IX	Carbonic anhydrase IX
CA-XII	Carbonic anhydrase XII
COX4-2	Cytochrome oxidase 4-2
CSCs	Cancer stem cells
CTLs	Cytotoxic T lymphocytes
DCs	Dendritic cells
Deptor	DEP-domain-containing mTOR-interacting protein
DLK1	Delta-like 1 Homologue
DNA-PKcs	DNA-dependent protein kinase, catalytic subunit
ECM	Extracellular matrix
EEF2K	Eukaryotic elongation factor 2 kinase
EIF2α	Eukaryotic initiation factor 2 alpha
EMT	Epithelial to mesenchymal transition
ENG	Endoglin
EphA2	Ephrin type-A receptor 2
ER	Endoplasmic reticulum
F6P	Fructose 6-phosphate
FBW7	F-box and WD repeat domain-containing 7
FDP	Fructose 1,6-bisphosphate
G6P	Glucose 6-phosphate
GLUTs	Glucose transporters
HA	Hyaluronic acid
HDAC	Histone deacetylases
HK2	Hexokinase 2
HIFs	Hypoxia-inducible factors
HREs	Hypoxia response elements
IGF2	Insulin-like growth factor
IKK	IκB kinase complex
IL-8	Interleukin 8
IRE1	Inositol-requiring protein 1
LDHA	Lactic dehydrogenase A
LEP	Leptin
LRP1	Low-density lipoprotein receptor-related protein 1
MCT1	Monocarboxylate transporter 1
mLST8	Mammalian lethal with Sec13 protein 8
mTOR	Mammalian target of rapamycin
MXI1	MAX interactor-1
NAD^+^	Nicotinamide adenine dinucleotide
NF-κB	Nuclear factor-κB
ODDD	Oxygen-dependent degradation domain
OXPHOS	Oxidative phosphorylation
p70S6K	P70S6 kinase
PDGF	Platelet-derived growth factor
PDH	Pyruvate dehydrogenase
PDK1	Pyruvate dehydrogenase kinase
PERK	Protein kinase RNA-like ER kinase
PFK1	Phosphofructokinase 1
PFKFB3	6-phosphofructo-2-kinase/fructose-2,6-biphosphatase 3
PGK1	Phosphoglycerate kinase 1
PIKKs	Phosphatidylinositol 3-kinase-related kinases
PKM	Pyruvate kinase M
PML	Promyelocytic leukemia protein
POU5F1	POU domain, class 5, transcription factor 1
PRAS40	Proline-rich AKT substrate 40 kDa
Raptor	Regulatory-associated protein of mTOR
REDD1	Regulated in development and DNA damage responses 1
ROS	Reactive oxygen species
TAD	Transactivation domains
TCA	Tricarboxylic acid cycle
TAMs	Tumor-associated macrophages
TFAM	Transcription factor A, mitochondrial
TGF	Transforming growth factor
TRAF6	Tumor necrosis factor receptor associated factor 6
Tregs	T cells regulatory
UPR	Uunfolded protein response
VEGF-A	Vascular endothelial growth factor-A
VHL	Von Hippel-Lindau
XBP1	X-box binding protein
